# Postoperative leukocyte counts as a surrogate for surgical stress response in matched robot- and video-assisted thoracoscopic surgery cohorts of patients: A preliminary report

**DOI:** 10.1007/s11701-024-01939-1

**Published:** 2024-04-17

**Authors:** Sidi Liu, Huichao Huang, Chunfang Zhang, Letao Chen, Xuelian Feng, Yaling Wu, Qing Xia, Xun Huang

**Affiliations:** 1 Infection Control Center, Xiangya Hospital of Central South University, 87 Xiangya Road, Changsha, 410008 China; 2 Department of Infectious Diseases, Xiangya Hospital of Central South University, Changsha, China; 3 Department of Thoracic Surgery, Xiangya Hospital of Central South University, Changsha, China; 4 Operating Room Department, Xiangya Hospital of Central South University, Changsha, China; 5 National Clinical Research Center for Geriatric Disorders, Xiangya Hospital of Central South University, Changsha, China; 6 Disease Prevention and Control Section, Anfu People’s Hospital, Jian, China; 7 Disease Prevention and Control Section, 921 Hospital of Joint Logistics Support Force, Changsha, China

**Keywords:** Postoperative leukocyte counts, Surgical stress response, Robot-assisted thoracoscopic surgery (RATS), Video-assisted thoracoscopic surgery (VATS), Non-small cell lung cancer (NSCLC)

## Abstract

The objective is to preliminary evaluated postoperative leukocyte counts as a surrogate for the surgical stress response in NSCLC patients who underwent RATS or VATS for further prospective analyses with proper assessment of surgical stress response and tissue trauma. We retrospectively analyzed patients with stageI-IIIA NSCLC who underwent RATS or VATS at a hospital between 8 May 2020 and 31 December 2021. Analysis of leukocytes (including neutrophils and lymphocytes) and albumin on postoperative days (PODs) 1 and 3 in patients with NSCLC treated with RATS or VATS after propensity score matching (PSM). In total, 1824 patients (565 RATS and 1259 VATS) were investigated. The two MIS groups differed significantly with regard to operative time (*p* < 0.001), chronic lung disease (*p* < 0.001), the type of pulmonary resection (*p* < 0.001), the excision site of lobectomy (*p* = 0.004), and histology of the tumor (*p* = 0.028). After PSM, leukocyte and neutrophil levels in the RATS group were lower than those in the VATS group on PODs 1 and 3, with those on POD 3 (*p* < 0.001) being particularly notable. While lymphocyte levels in the RATS group were significantly lower than those in the VATS group only at POD 1 (*p* = 0.016). There was no difference in albumin levels between the RATS and VATS groups on PODs 1 and 3. The surgical stress response and tissue trauma was less severe in NSCLC patients who underwent RATS than in those who underwent VATS, especially reflected in the neutrophils of leukocytes.

## Introduction

Minimally invasive surgery (MIS) techniques, currently considered the gold standard in the treatment of non-small cell lung cancer (NSCLC) in China and worldwide, are represented mainly by robot-assisted thoracoscopic surgery (RATS) and video-assisted thoracoscopic surgery (VATS). Studies have shown that RATS improves visibility with a three-dimensional visibility stereoscopic view and magnified field of view compared to VATS; similarly, it allows for greater precision owing to multijoint instrumentation and reduction in human hand tremors [1, 2]. Moreover, the clear vision and precise accuracy of RATS may reduce the surgical stress response and tissue trauma of the patient.

Currently, postoperative surgical stress response has been measured after different surgical procedures (esophagus, colon, gastric, liver, etc.) [3–5] , but little is known about that in NSCLC patients after RATS and VATS. The stress response to surgery is a complex neuroendocrine-metabolic and inflammatory-immune process [6]. Recovery after surgery has been tracked by serial measurement of a number of blood markers, such as cortisol, interleukin 6 (IL-6), Leukocyte, and C-reactive protein (CRP) [7]. Leukocyte, especially neutrophil, play a central role in the systemic inflammatory response after surgery and also be used as a surrogate for surgical stress response. Therefore, we conducted this preliminary study by propensity score-matched analysis to compare leukocytes on postoperative days (PODs) in patients with NSCLC treated with RATS or VATS for further prospective analyses with proper assessment of surgical stress response and tissue trauma.

## Patients and methods

### Design

This is a retrospective and comparative cohort study that was conducted at Xiangya Hospital of Central South University. We included all patients aged ≥ 18 years who underwent RATS or VATS for stageI-IIIA non-small cell lung cancer between 8 May 2020 and 31 December 2021. Leukocytes and albumin were tested preoperatively as well as on PODs 1 and 3. The exclusion criteria were open thoracotomy, preoperative infection, HIV infection, history of malignant blood disease, autoimmune disease, immunosuppression or corticosteroid treatment, or history of radiotherapy or chemotherapy within the last 6 months. Eligible patients were monitored until discharge from the hospital and were followed up through the outpatient clinic or received a telephone follow-up 30 days after surgery to track their postoperative pneumonia (POP) outcome.

## Definitions

POP was defined as pneumonia occurring within 30 days postsurgery in patients with no evidence of pneumonia preoperatively, and the diagnosis of pneumonia required patients to meet two major criteria plus one minor criterion or one major criterion plus three minor criteria. The major criteria were fever (> 38 °C) with no other recognizable cause and abnormal radiographic findings (new or progressive and persistent infiltrate, consolidation, or opacity). The minor criteria were leukopenia < 4.0 × 10^9^ cells/L or leukocytosis > 12.0 × 10^9^ cells/L, a new rise in CRP level, increase and modification of the expectorate toward a purulent appearance. Three experienced clinicians, namely, two infection prevention and control specialists and a cardiothoracic surgeon, worked together to diagnose POP.

## Surgical procedures

RATS or VATS was performed with the patient under general anesthesia and in the lateral decubitus position. RATS was performed using the da Vinci (Xi) surgical platform with a four-arm approach. Usually, RATS has four surgical incisions, while VATS has two. The surgical method, including the type of pulmonary resection and whether lymph node dissection or sampling was necessary, was selected according to the results of intraoperative frozen section examination and preoperative examination. For patients requiring lobectomy or segmentectomy, anatomic resection was performed.

## Data collection

The NSCLC patients were divided into the RATS group or VATS group according to the surgical approach. The following preoperative, intraoperative, and postoperative variables were reviewed for the two groups: sex, age, smoking history, smoking index (cigarettes per year), chronic disease (chronic lung disease, hypertension, diabetes, coronary heart disease), American Society of Anesthesiologists (ASA) score, type of pulmonary resection, excision site of lobectomy, histology of the tumor, operative time, intraoperative blood loss, POP, and postoperative hospitalization days.

The leukocyte (including neutrophil and lymphocyte) and albumin counts analyzed in this study were those determined from blood drawn on the day of admission and on PODs 1 and 3. The leukocyte was obtained via complete blood count analysis, and albumin was measured via liver function analysis. The operative time was calculated as the interval between anesthesia induction and closure of the incision.

## Statistical analysis

Data were entered and analyzed using SPSS version 27. Qualitative data are summarized as numbers and percentages, and quantitative data are reported as medians (Q25/Q75). Qualitative data were analyzed by Pearson's chi-squared test, and quantitative data were analyzed using the Wilcoxon rank-sum test (also known as the Mann‒Whitney U test) to assess differences in these variables between the RATS group and VATS group before propensity score matching (PSM). A propensity score-matched analysis was performed using a multivariable logistic regression model based on preoperative and perioperative covariates: sex, age, smoking, smoking index, chronic lung disease, hypertension, diabetes, coronary heart disease, ASA, histology of the tumor, type of pulmonary resection, and excision site of lobectomy. Pairs of patients who underwent RATS or VATS were derived using 1:1 greedy nearest neighbor matching within a PS score of 0.02. Quantitative data are reported as the median (Q25/Q75), and statistical analysis was performed using the Wilcoxon signed-rank test for paired samples after PSM. A *p* value of < 0.05 was considered to show statistical significance.

## Results

### Patient characteristics

**Fig. 1:**
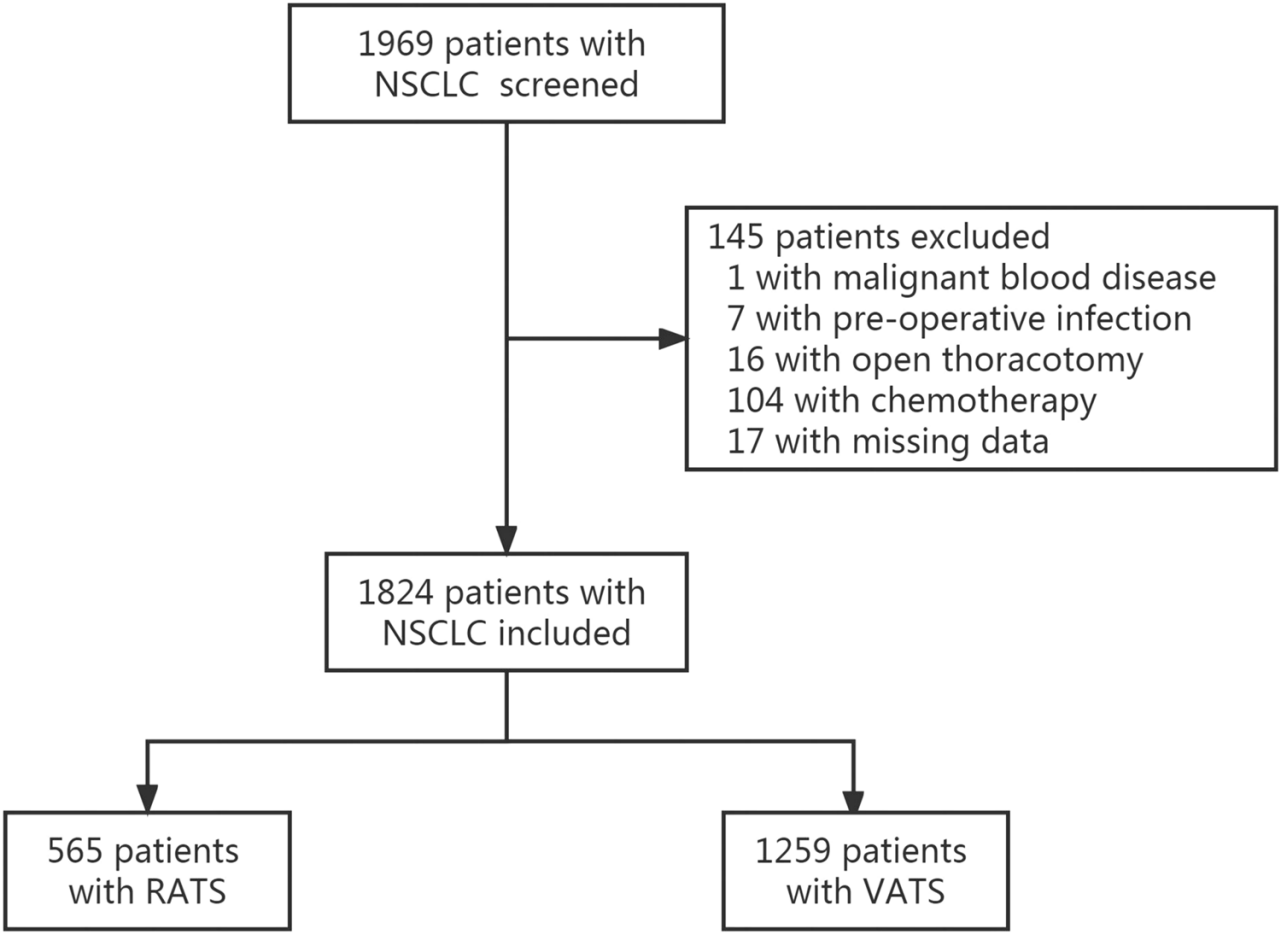
Flow chart of the study. NSCLC, non-small cell lung cancer; RATS: robot-assisted thoracoscopic surgery; VATS: video-assisted thoracoscopic surgery

From 8 May 2020 to 31 December 2021, 1969 patients with NSCLC were screened, among whom 145 were excluded and 1824 were deemed to meet the inclusion criteria (Fig. 1).

The clinical characteristics of these 1824 patients are shown in Table 1. Of these, 994 (54.5%) were female, 761 (41.7%) were aged 50–59 years, 590 (32.3%) had a history of smoking, and 519 (28.5%) experienced chronic lung disease. A total of 1259 (69.0%) patients and 565 (31.0%) patients with NSCLC voluntarily chose VATS and RATS, respectively. Lobectomy was performed in 1246 (68.3%) patients, of which 481 (38.6%) had the right middle lobe removed. A total of 1657 (90.8%) patients with NSCLC had adenocarcinoma according to postoperative pathology records.

**Table 1:** Clinical characteristics of 1824 patients with NSCLC

Clinical characteristics	Data
Sex, n (%)	
Male	830 (45.5)
Female	994 (54.5)
Age groups (years), n (%)	
< 50	330 (18.1)
50–59	761 (41.7)
60–69	540 (29.6)
70–79	187 (10.3)
≥ 80	6 (0.3)
Smoking, n (%)	
Yes	590 (32.3)
Smoking index (cigarettes per year), n (%)	
≤ 400	190 (32.2)
> 400	400 (67.8)
Chronic disease	
Chronic lung disease ^1^, n (%)	
Yes	519 (28.5)
Hypertension, n (%)	
Yes	424 (23.2)
Diabetes, n (%)	
Yes	158 (8.7)
Coronary heart disease, n (%)	
Yes	95 (5.2)
ASA, n (%)	
1	33 (1.8)
2	772 (42.3)
3	985 (54.0)
4	34 (1.9)
Histology, n (%)	
Adenocarcinoma	1657 (90.8)
Squamous cell carcinoma	138 (7.6)
Large cell carcinoma	1 (0.1)
Unclassified NSCLC ^2^	28 (1.5)
Approaches of surgery, n (%)	
RATS	565 (31.0)
VATS	1259(69.0)
Type of pulmonary resection, n (%)	
Lobectomy	1246 (68.3)
Segment resection	337 (18.5)
Wedge resection	121 (6.6)
Lobectomy + wedge resection	41 (2.2)
Lobectomy + segment resection	7 (0.4)
Segment resection + wedge resection	72 (3.9)
Excision site of lobectomy ^3^, n (%)	
Left upper lobe	251 (20.1)
Left lower lobe	183 (14.7)
Right upper lobe	481 (38.6)
Right middle lobe	116 (9.3)
Right lower lobe	194 (15.6)
Multiple lobes	21 (1.7)

*RATS *robot-assisted thoracoscopic surgery, *VATS* video-assisted thoracoscopic surgery, *NSCLC *non-small cell lung cancer, *ASA *American society of anesthesiologists

^1^ Chronic lung disease included chronic obstructive pulmonary disease, chronic bronchitis, asthma, and bronchiectasis

^2^ Unclassified NSCLC is when the tumor is NSCLC, but no further classification has been performed

^3^ Excision site of lobectomy is for 1246 patients who only underwent lobectomy

## Preoperative, intraoperative, and postoperative variable analysis

Patients in the RATS group suffered from prolonged operative time (*p* < 0.001) compared to the VATS group. The two MIS groups differed significantly with regard to chronic lung disease (*p* < 0.001), the type of pulmonary resection (*p* < 0.001), the excision site of lobectomy (*p* = 0.004), and histology of the tumor (*p* = 0.028). POP was observed in 39 (6.9%, 39/565) patients and 87 (6.9%, 87/1259) patients after RATS and VATS, respectively (*p* > 0.05) (Table 2).

**Table 2:** Preoperative, intraoperative, and postoperative variable analysis between VATS and RATS

Variables	RATS (n = 565)	VATS (n = 1259)	*p*
Preoperative			
Sex, n (%)			0.410
Male	249 (44.1)	581 (46.1)	
Female	316 (55.9)	678 (53.9)	
Age groups (in years), n (%)			
< 50	116 (20.5)	214 (17.0)	0.233
50–59	237 (41.9)	524 (41.6)	
60–69	153 (27.1)	387 (30.7)	
70–79	56 (9.9)	131 (10.4)	
≥ 80	3 (0.5)	3 (0.2)	
Smoking, n(%)			0.343
Yes	174 (30.8)	416 (33.0)	
Smoking index (cigarettes per year), n (%)			0.083
≤ 400	65 (37.4)	125 (30.0)	
> 400	109 (62.6)	291 (70.0)	
Chronic disease, n (%)			
Chronic lung disease			< 0.001***
Yes	240 (42.5)	279 (22.2)	
Hypertension			0.318
Yes	123 (21.8)	301 (23.9)	
Diabetes			0.992
Yes	49 (8.7)	109 (8.7)	
Coronary heart disease			0.558
Yes	32 (5.7)	63 (5.0)	
ASA, n (%)			0.286
1	13 (2.3)	20 (1.6)	
2	230 (40.7)	542 (43.1)	
3	315 (55.8)	670 (53.2)	
4	7 (1.2)	27 (2.1)	
Perioperative			
Type of pulmonary surgery, n (%)			< 0.001***
Lobectomy	266 (47.1)	980 (77.8)	
Segment resection	175 (31.0)	162 (12.9)	
Wedge resection	63 (11.2)	58 (4.6)	
Lobectomy + wedge resection	1 (0.2)	40 (3.2)	
Lobectomy + segment resection	1 (0.2)	6 (0.5)	
Segment resection + wedge resection	59 (10.4)	13 (1.0)	
Excision site of lobectomy, n (%)			0.004**
Left upper lobe	34 (12.8)	217 (22.1)	
Left lower lobe	37 (13.9)	146 (14.9)	
Right upper lobe	117 (44.0)	364 (37.1)	
Right middle lobe	33 (12.4)	83 (8.5)	
Right lower lobe	43 (16.2)	151 (15.4)	
Multiple lobes	2 (0.8)	19 (1.9)	
Histology of the tumor, n (%)			0.028*
Adenocarcinoma	530 (93.8)	1127 (89.5)	
Squamous carcinoma	28 (5.0)	110 (8.7)	
Large cell carcinoma	0	1(0.1)	
Uncategorized NSCLC	7 (1.2)	21 (1.7)	
Operative time (minutes), median (Q25/Q75)	125(100, 160)	99(80, 130)	< 0.001***
Intraoperative blood loss (mL), median (Q25/Q75)	50 (50,100)	50 (50,100)	0.135
Postoperative			
POP, n (%)	39 (6.9)	87 (6.9)	1.000
Postoperative hospital days (days), median (Q25/Q75)	5 (4,6)	5 (4,6)	0.406

^*^: *p* < 0.05; **: *p* < 0.01; ***: *p* < 0.001; *RATS* robot-assisted thoracoscopic surgery, *VATS* video-assisted thoracoscopic surgery, *POP* postoperative pneumonia

## Leukocyte and albumin levels after PSM

After PSM analysis, there were 76 matched pairs in the two groups. After PSM, no differences in the preoperative baseline levels of leukocytes (including neutrophils and lymphocytes) or albumin were observed between the RATS group and VATS group.

**Fig. 2:**
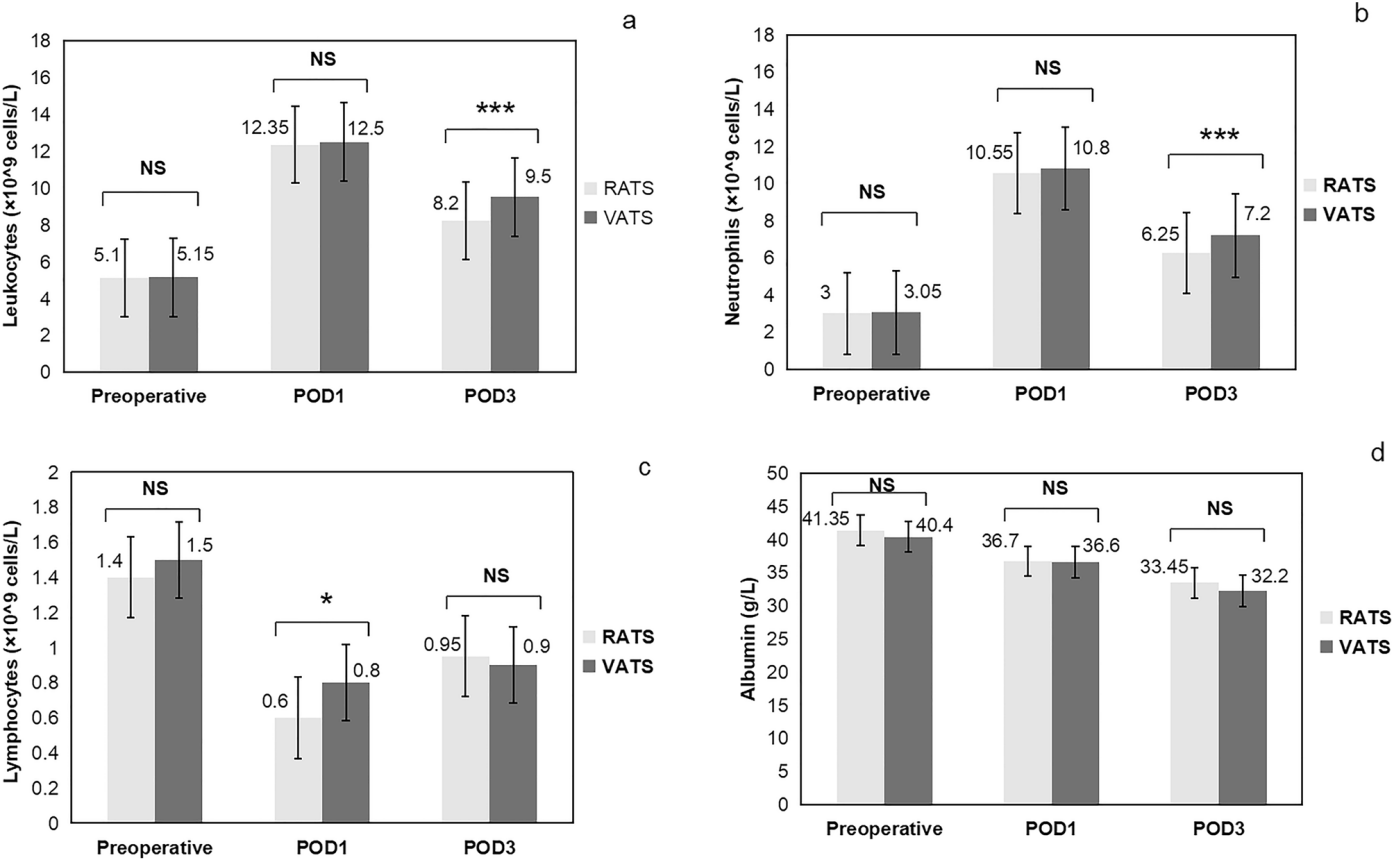
Leukocyte, neutrophil, lymphocyte and albumin levels was measured preoperatively and on PODs 1 and 3 of patients in the RATS and VATS group after PSM.**a** The mean leukocyte counts; **b** The mean neutrophil counts; **c** The mean lymphocyte counts; **d** The mean albumin counts; RATS: robot-assisted thoracoscopic surgery; VATS: video-assisted thoracoscopic surgery; PODs: postoperative days; NS: no significance; *: *p* < 0.05; ***: *p* < 0.001

After PSM, increased concentrations of leukocytes were found on PODs 1 and 3 in both the RATS and VATS groups compared to their respective leukocyte levels on preoperative, and the highest leukocyte level occurred on POD 1. The leukocyte levels in the RATS group were lower than those in the VATS group on PODs 1 and 3, with those at POD 3 (*p* < 0.001) being particularly notable (Fig. 2a). Similarly, neutrophil levels were increased on PODs 1 and 3 in both the RATS and VATS groups compared to their respective neutrophil levels on preoperative, and the neutrophil level was highest on POD 1. Neutrophils in the RATS group were significantly lower than those in the VATS group at POD 3 (*p* < 0.001) (Fig. 2b). In contrast, compared to lymphocyte levels on preoperative, reduced lymphocyte concentrations were found on PODs 1 and 3 in both groups, with the lymphocyte levels being the lowest at POD 1. The lymphocyte level in the RATS group was significantly lower than that in the VATS group only at POD 1 (*p* = 0.016) (Fig. 2c).

After PSM, albumin concentrations at PODs 1 and 3 decreased in both the RATS and VATS groups compared with albumin levels on preoperative, with the lowest albumin levels at POD 3. Although there was no difference in albumin levels between the RATS and VATS groups on PODs 1 and 3, albumin was slightly lower in the VATS group than in the RATS group (Fig. 2d).

## Discussion

MIS for the treatment of NSCLC has rapidly spread during the last decade as a consequence of numerous investigators have proven its safety and effectiveness [8, 9]. The advantages of MIS compared to open thoracic surgery are less trauma, less pain, fewer complications (including POP) and a better quality of life [10–13]. According to guidelines, VATS or RATS should be strongly considered for patients with no anatomic or surgical contraindications [14]. Although VATS is the first non-rib-spreading thoracic procedure described, the introduction of RATS is undoubtedly the most recent significant addition to the field of thoracic surgery. Currently, less consensus has been achieved on whether to choose VATS or RATS for patients with NSCLC. Studies have shown no difference in postoperative complications, the conversion rate to open surgery, mortality, overall survival and disease-free survival between RATS and VATS as minimally invasive surgery techniques [15, 16], but RATS is a newer technique for MIS that has addressed some of the shortcomings of VATS with 3D visibility and mechanical wrists [2, 17, 18]. The clear vision and precise accuracy of RATS may reduce tissue trauma in the patient. From this point of view, one of the hypothesized advantages of the RATS approach could be considered the reduced surgical stress response.

The surgical stress response plays a key role in surgical outcomes. A mild stress response can improve the adaptability of the human body, while an excessive stress response exacerbates functional impairment of the human body [19]. Lobectomy is the gold standard resection for NSCLC in patients who can tolerate anatomical lung resection, so RATS and VATS primarily injure lung tissue and thus can cause postoperative complications of POP in patients with NSCLC. Our results showed that the incidence of POP was 6.9% in both the RATS group and VATS group. There was no difference in the occurrence of POP between the two minimally invasive surgical approaches [20, 21]. Therefore, we quantified tissue trauma in both the RATS group and VATS group by selecting leukocytes and albumin as indicators of the surgical stress response.

Leukocytes, a type of proinflammatory cytokines, are produced by injured tissue as a direct consequence of trauma. Leukocytes include granulocytes (neutrophils, eosinophils and basophils), lymphocytes (T cells, B cells and NK cells), and monocytes (macrophages and dendritic cells). In general, the total leukocyte count is relatively stable and increases after surgery or in the presence of inflammation, so leukocytes are used to assess the degree of postsurgical trauma and surgical stress response. Mari et al. [22] integrated leukocytes as well as IL-6 and CRP as indicators to compare the surgical stress response level in two groups of patients who underwent laparoscopic right hemi-colectomy with intracorporeal or extracorporeal anastomosis. Likewise, leukocytes were used as one of the indicators to assess the surgical stress response of different perioperative management measures in patients with gastric cancer complicated by type 2 diabetes mellitus [23]. In addition, leukocytes are an important marker of inflammation. Therefore, it is reasonable for us to choose leukocytes as the primary indicator of the surgical stress response to assess tissue trauma by RATS or VATS.

At present, less is known about the differences in surgical stress response in NSCLC patients who underwent RATS and VATS. Herein, we preliminary evaluated postoperative leukocyte counts as a surrogate for the surgical stress response in NSCLC patients who underwent RATS or VATS in the study. We selected leukocytes, whose production is a direct consequence of tissue trauma, as the primary indicator of the surgical stress response. In our study, preoperative and intraoperative variables that may influence leukocytes or albumin, including demographic information as well as the type of pulmonary resection, excision site of lobectomy, and histology of the tumor, were adjusted for baseline differences and selection bias by PSM. After PSM, there was no difference in the preoperative baseline levels of leukocytes (including neutrophils and lymphocytes) and albumins between the RATS and VATS groups.

After PSM, we found that the leukocyte levels of the patients peaked at POD 1 in the two MIS groups and gradually returned to baseline leukocyte levels from POD 3. Moreover, the patients in the RATS group had a significantly faster return to preoperative baseline leukocyte levels than those in the VATS group (Fig. 2a). The above findings are most evident in neutrophils (Fig. 2b), as a component of leukocytes. Lung cancer resection is a strong stimulus to the body, so the most pronounced surgical stress response occurs on the first postoperative day, followed by a gradual return to normal, and this trend of the surgical stress response is also reflected in other surgeries [24]. We also found that the level of lymphocytes was lower in the RATS group than in the VATS group at POD 1. This may be related to lymph node clearance. Adequate lymphadenectomy is a milestone in the treatment of NSCLS [25]. Studies revealed that RATS could achieve more lymph node dissections than VATS [26, 27] which indicated that the lymph node dissection of RATS was more thorough.

Albumin, as a negative acute phase protein and a nutritional biomarker, usually decreases after surgery due to surgical stress and increased capillary leakage [28]. During the process of the surgical stress response, capillary vascular endothelial cells are destroyed due to the release of a large number of inflammatory factors in the blood, resulting in increased capillary permeability of the whole body and hypoproteinemia caused by leakage of intravascular albumin. Extravasation of plasma albumin from the vessel wall occurs at the beginning of surgery and peaks at POD 2 or POD 3 [28]. Albumin levels in the two MIS groups showed a continuous decrease 3 days postoperatively in our study. Although there was no difference in albumin levels between the two MIS groups, the level in the VATS group was slightly lower than that in the RATS group.

In this study, RATS prolonged operating time but not the postoperative time and did not increase intraoperative blood loss or the incidence of postoperative POP. However, previous studies have reported the advantage of RATS over VATS in reducing hospital stays as well as operative time [29, 30]. Because RATS has more surgical incisions than VATS in the study, which may have resulted in a longer operation time.

Limilations to this retrospective study that no additional indicators of surgical stress were tested, including tumor necrosis factor-α (TNF-α), IL-6, IL-10 and CRP. Because this is a retrospective study, patients undergoing RATS or VATS are usually tested for routine postoperative blood and liver function for the patient's financial benefit, unless the patient presents with signs of infection, in which case they are tested for other inflammatory markers, such as TNF-α, IL-6, IL-10 and CRP.

## Conclusions

Our study demonstrates that no difference in the incidence of POP caused by RATS and VATS as minimally invasive surgical approaches. However, RATS, a newer technique for MIS with 3D visibility and mechanical wrists, caused less surgical stress response and tissue trauma than VATS in NSCLC patients, and this was especially reflected in the leukocytes on POD 3. In this study, we preliminary evaluated postoperative leukocyte counts as a surrogate for the surgical stress response in NSCLC patients who underwent RATS or VATS for further prospective analyses with proper assessment of surgical stress response and tissue trauma.

## Data Availability

The raw data supporting the conclusions of this article will be made available by the. authors, without undue reservation.

## Abbreviations

ASA: American society of anesthesiologists
CRP: C-reactive protein
IL-6: Interleukin 6
MIS: Minimally invasive surgery
NSCLC: Non-small cell lung cancer
PODs: Postoperative days
POP: Postoperative pneumonia
PSM: Propensity score matching
RATS: Robot-assisted thoracoscopic surgery
TNF-α: Tumor necrosis factor-α
VATS: Video-assisted thoracoscopic surgery

## References

[1] Raman V, Christopher JK, Jawitz OK et al (2020) Robot- vs video-assisted thoracoscopic lobectomy for early lung cancer. JNCI Cancer Spectr. 10.1093/jncics/pkaa031 33215061 10.1093/jncics/pkaa031 PMC7660032

[2] Kent M, Wang T, Whyte R et al (2014) Open, video-assisted thoracic surgery, and robotic lobectomy: review of a national database. Ann Thorac Surg 97:236–244. 10.1016/j.athoracsur.2013.07.117 24090577 10.1016/j.athoracsur.2013.07.117

[3] Sakuramoto S, Kikuchi S, Futawatari N et al (2009) Laparoscopy-assisted pancreas- and spleen-preserving total gastrectomy for gastric cancer as compared with open total gastrectomy. Surg Endosc 23:2416–2423. 10.1007/s00464-009-0371-0 19266232 10.1007/s00464-009-0371-0

[4] Watt DG, Horgan PG, McMillan DC (2015) Routine clinical markers of the magnitude of the systemic inflammatory response after elective operation: a systematic review. Surgery 157:362–380. 10.1016/j.surg.2014.09.009 25616950 10.1016/j.surg.2014.09.009

[5] Franke A, Lante W, Fackeldey V et al (2005) Pro-inflammatory cytokines after different kinds of cardio-thoracic surgical procedures: is what we see what we know? Eur J Cardiothorac Surg 28:569–575. 10.1016/j.ejcts.2005.07.007 16135408 10.1016/j.ejcts.2005.07.007

[6] Li H, Li J, Hao C et al (2023) Effects of anesthetic depth on perioperative T lymphocyte subsets in patients undergoing laparoscopic colorectal cancer surgery: a prospective, parallel-controlled randomized trial. BMC Anesthesiol 23:165. 10.1186/s12871-023-02129-6 37189020 10.1186/s12871-023-02129-6 PMC10184475

[7] Cusack B, Buggy DJ (2020) Anaesthesia, analgesia, and the surgical stress response. BJA Educ 20:321–328. 10.1016/j.bjae.2020.04.006 33456967 10.1016/j.bjae.2020.04.006 PMC7807970

[8] Dziedzic R, Marjanski T, Binczyk F et al (2018) Favourable outcomes in patients with early-stage non-small-cell lung cancer operated on by video-assisted thoracoscopic surgery: a propensity score-matched analysis. Eur J Cardio-Thorac 54:547–553. 10.1093/ejcts/ezy101 10.1093/ejcts/ezy101 29547899

[9] Boffa DJ, Kosinski AS, Furnary AP et al (2018) Minimally invasive lung cancer surgery performed by thoracic surgeons as effective as thoracotomy. J Clin Oncol 36:2378–2385. 10.1200/JCO.2018.77.8977 29791289 10.1200/JCO.2018.77.8977

[10] Simonsen DF, Sogaard M, Bozi I et al (2015) Risk factors for postoperative pneumonia after lung cancer surgery and impact of pneumonia on survival. Resp Med 109:1340–1346. 10.1016/j.rmed.2015.07.008 10.1016/j.rmed.2015.07.008 26209227

[11] Novellis P, Maisonneuve P, Dieci E et al (2021) Quality of life, postoperative pain, and lymph node dissection in a robotic approach compared to VATS and OPEN for early stage lung cancer. J Clin Med 10:1687. 10.3390/jcm10081687 33920023 10.3390/jcm10081687 PMC8071041

[12] Lee JY, Jin SM, Lee CH et al (2011) Risk factors of postoperative pneumonia after lung cancer surgery. J Korean Med Sci 26:979–984. 10.3346/jkms.2011.26.8.979 21860545 10.3346/jkms.2011.26.8.979 PMC3154353

[13] Van APT, Ayez N, Akkersdijk GP et al (2020) Postoperative pain after lobectomy: robot-assisted, video-assisted and open thoracic surgery. J Robot Surg 14:131–136. 10.1007/s11701-019-00953-y 30927155 10.1007/s11701-019-00953-y

[14] Mangiameli G, Cioffi U, Testori A (2022) Lung cancer treatment: from tradition to innovation. Front Oncol 12:858242. 10.3389/fonc.2022.858242 35692744 10.3389/fonc.2022.858242 PMC9184755

[15] Wu H, Jin R, Yang S et al (2021) Long-term and short-term outcomes of robot versus video-assisted anatomic lung resection in lung cancer: a systematic review and meta-analysis. Eur J Cardiothorac Surg 59:732–740. 10.1093/ejcts/ezaa426 33367615 10.1093/ejcts/ezaa426

[16] Ma J, Li X, Zhao S et al (2021) Robot-assisted thoracic surgery versus video-assisted thoracic surgery for lung lobectomy or segmentectomy in patients with non-small cell lung cancer: a meta-analysis. BMC Cancer 21:498. 10.1186/s12885-021-08241-5 33941112 10.1186/s12885-021-08241-5 PMC8094485

[17] Zhang L, Gao S (2015) Robot-assisted thoracic surgery versus open thoracic surgery for lung cancer: a system review and meta-analysis. Int J Clin Exp Med 8:17804–17810. PMID: 26770372; PMCID: PMC4694272. PMC4694272 26770372

[18] Veronesi G, Novellis P, Voulaz E et al (2016) Robot-assisted surgery for lung cancer: state of the art and perspectives. Lung Cancer 101:28–34. 10.1016/j.lungcan.2016.09.004 27794405 10.1016/j.lungcan.2016.09.004

[19] Lin Y, Zhang G, Wang Y et al (2018) Prognostic evaluation of child patients with infectious encephalitis through AEEG and REEG. Exp Ther Med 16:5243–5247. 10.3892/etm.2018.6882 30542480 10.3892/etm.2018.6882 PMC6257196

[20] Xie B, Sun X, Qin Y et al (2018) Short-term outcomes of typical versus atypical lung segmentectomy by minimally invasive surgeries. Thoracic cancer 10:1812–1818. 10.1111/1759-7714.13152 10.1111/1759-7714.13152 PMC6718014 31373437

[21] Hu X, Wang M (2019) Efficacy and safety of Robot-assisted Thoracic Surgery (RATS) Compare with Video-assisted Thoracoscopic Surgery (VATS) for lung lobectomy in patients with non-small cell lung cancer. Comb Chem High Throughput Screen 22:169–178. 10.2174/1386207322666190411113040 30973106 10.2174/1386207322666190411113040

[22] Mari GM, Crippa J, Costanzi ATM et al (2018) Intracorporeal anastomosis reduces surgical stress response in laparoscopic right hemicolectomy: a prospective randomized trial. Surg Laparo Endo Per 28:77–81. 10.1097/SLE.0000000000000506 10.1097/SLE.0000000000000506 29360701

[23] Chen JX, Lin R, Fan X et al (2022) Effect of enhanced recovery after surgery on surgical stress response in patients with gastric cancer complicated with type 2 diabetes mellitus. Natl Med J China 102:847–852 10.3760/cma.j.cn112137-20211130-02673 35330577

[24] Milone M, Desiderio A, Velotti N et al (2021) Surgical stress and metabolic response after totally laparoscopic right colectomy. Sci Rep 11:9652. 10.1038/s41598-021-89183-7 33958669 10.1038/s41598-021-89183-7 PMC8102592

[25] Aljaafari D, Ishaque N (2022) Thymectomy in myasthenia gravis: a narrative review. Saudi J Med Med Sci 10:97–104. 10.4103/sjmms.sjmms_80_22 35602390 10.4103/sjmms.sjmms_80_22 PMC9121707

[26] Haruki T, Takagi Y, Kubouchi Y et al (2021) Comparison between robot-assisted thoracoscopic surgery and video-assisted thoracoscopic surgery for mediastinal and hilar lymph node dissection in lung cancer surgery. Interact Cardiovasc Thorac Surg 33:409–417. 10.1093/icvts/ivab112 34297835 10.1093/icvts/ivab112 PMC8691695

[27] Kneuertz PJ, Cheufou DH, D’Souza DM et al (2019) Propensity-score adjusted comparison of pathologic nodal upstaging by robotic, video-assisted thoracoscopic, and open lobectomy for non-small cell lung cancer. J Thorac Cardiov Sur 158:1457–1466. 10.1016/j.jtcvs.2019.06.113 10.1016/j.jtcvs.2019.06.113 31623811

[28] Fleck A, Raines G, Hawker F et al (1985) Increased vascular permeability: a major cause of hypoalbuminaemia in disease and injury. Lancet 1:781–784. 10.1016/s0140-6736(85)91447-3 2858667 10.1016/s0140-6736(85)91447-3

[29] Shen C, Li J, Che G (2021) Video-assisted thoracic surgery vs. thoracotomy for the treatment in patients with esophageal leiomyoma: a systematic review and meta-analysis. Frontiers surgery. 8:809253. 10.3389/fsurg.2021.809253 10.3389/fsurg.2021.809253 PMC8786717 35087862

[30] Huang J, Tian Y, Zhou QJ et al (2021) Comparison of perioperative outcomes of robotic-assisted versus video-assisted thoracoscopic right upper lobectomy in non-small cell lung cancer. Trans lung cancer res 10:4549–4557. 10.21037/tlcr-21-960 10.21037/tlcr-21-960 PMC8743526 35070760

